# Racial/ethnic disparities in opioid overdose prevention: comparison of the naloxone care cascade in White, Latinx, and Black people who use opioids in New York City

**DOI:** 10.1186/s12954-023-00736-7

**Published:** 2023-02-25

**Authors:** Maria R. Khan, Lee Hoff, Luther Elliott, Joy D. Scheidell, John R. Pamplin, Tarlise N. Townsend, Natalia M. Irvine, Alex S. Bennett

**Affiliations:** 1 Department of Population Health, New York University Grossman School of Medicine, New York, NY USA; 2 Center for Drug Use and HIV/HCV Research, New York University School of Global Public Health, New York, NY USA; 3 Center for Opioid Epidemiology and Policy, New York University Grossman School of Medicine, New York, NY USA; 4 Department of Social and Behavioral Sciences, New York University School of Global Public Health, New York, NY USA; 5 Columbia University Mailman School of Public Health, Department of Epidemiology, New York, NY USA; 6 Center for Anti-Racism, Social Justice, and Public Health, New York University School of Global Public Health, New York, NY USA

**Keywords:** Naloxone care, Prevention, Racial/ethnic disparities

## Abstract

**Background:**

Drug overdose mortality is rising precipitously among Black people who use drugs. In NYC, the overdose mortality rate is now highest in Black (38.2 per 100,000) followed by the Latinx (33.6 per 100,000) and white (32.7 per 100,000) residents. Improved understanding of access to harm reduction including naloxone across racial/ethnic groups is warranted.

**Methods:**

Using data from an ongoing study of people who use illicit opioids in NYC (*N* = 575), we quantified racial/ethnic differences in the naloxone care cascade.

**Results:**

We observed gaps across the cascade overall in the cohort, including in naloxone training (66%), current possession (53%) daily access during using and non-using days (21%), 100% access during opioid use (20%), and complete protection (having naloxone and someone who could administer it present during 100% of opioid use events; 12%). Naloxone coverage was greater in white (training: 79%, possession: 62%, daily access: 33%, access during use: 27%, and complete protection: 13%, respectively) and Latinx (training: 67%, possession: 54%, daily access: 22%, access during use: 24%, and complete protection: 16%, respectively) versus Black (training: 59%, possession: 48%, daily access:13%, access during use: 12%, and complete protection: 8%, respectively) participants. Black participants, versus white participants, had disproportionately low odds of naloxone training (OR 0.40, 95% CI 0.22–0.72). Among participants aged 51 years or older, Black race (versus white, the referent) was strongly associated with lower levels of being trained in naloxone use (OR 0.20, 95% CI 0.07–0.63) and having 100% naloxone access during use (OR 0.34, 95% CI 0.13–0.91). Compared to white women, Black women had 0.27 times the odds of being trained in naloxone use (95% CI 0.10–0.72).

**Conclusions:**

There is insufficient protection by naloxone during opioid use, with disproportionately low access among Black people who use drugs, and a heightened disparity among older Black people and Black women.

## Introduction

New York City (NYC) drug overdose deaths have increased precipitously since the start of the COVID-19 pandemic, with the rate of overdose having increased to 39.4 per 100,000 NYC residents in 2021 from 31.6 in 2020. In NYC, New Yorkers who were Black and/or Latinx had largest absolute increase from 2020 to 2021 (an increase in the rate of approximately 13.5 per 100,000 in each group). The overdose mortality rate remains disproportionately highest in the NYC Black ( 53.5 per 100,000) and Latinx (49.2 per 100,000) populations compared with the  white (36.2 per 100,000) population [1]. The top three hardest-hit NYC neighborhoods are in predominantly Black and Latinx neighborhoods in the Bronx, where drug overdose rates exceeded even the hardest-hit US states [1]. The alarming increase in overdose mortality among Black populations in NYC is observed nationally, with recent trend analyses indicating that the increase in opioid overdose mortality from 2010 to 2018 was greater among Black than white US residents [2]. Improved understanding of access to life-saving harm reduction programming overall, and in racial/ethnic minority groups in particular, is a critical public health priority.

Over the past 20 years, the number of opioid overdose prevention programs offering naloxone has expanded markedly [3]. Yet, even in NYC, where city and state departments of health have led concerted and well-funded efforts to saturate the city with naloxone, considerable limitations and disparities in coverage exist [4, 5]. Black and Latinx groups, less likely to receive access to quality healthcare in general, may also experience reduced access to naloxone [6–10]. These groups may face financial barriers to care [11] and experiences of medical and systems mistrust as a result of experiences of discriminatory, stigmatizing, and poor quality care [12–14]. Even if Black and Latinx individuals receive naloxone training and take home a naloxone kit, other barriers may hinder consistent access to naloxone during use [15]. Given Black and Latinx people experience disproportionate police contact and incarceration [16], they may fear that carrying naloxone may prompt searches for opioids and result in arrest and detainment [10, 17]. People who use opioids also may face reprisal and stigmatization [18] from family and friends [17], who may be cued to opioid use by the presence of a naloxone kit [17]. Against the backdrop of systemic racism, some have voiced concerns that the presence of syringe service programs and other agencies offering naloxone would lead to further stigmatization of minority communities [19, 20]. Racist stereotyping of people who use drugs [19, 20], with a lack of cultural competence in some naloxone service delivery programming [21] may contribute to reduced naloxone uptake [10].

There is empirical evidence of racial/ethnic disparities in access [11, 22], training and administration of naloxone [5, 23–27]. One study indicated Black people who inject drugs but who do not access services at a syringe service program are among the least likely group to possess naloxone, suggesting the need for targeted outreach to this group [28]. However, results are not universally consistent, with some evidence suggesting improved coverage among Black or African American residents [29]. Additional research on levels of access to naloxone and variation in coverage by race/ethnicity is warranted.

To address this research gap, using data collected from a racially and ethnically diverse cohort of people who use illicit opioids in NYC, the current study describes racial/ethnic differences in engagement in the naloxone care cascade. The cascade maps the steps through which an opioid user must pass in order to be protected by naloxone during opioid use. The steps include being trained in naloxone administration, possessing naloxone, and having both naloxone and someone present to administer it, if needed, when using opioids [30]. In this paper, we compare the naloxone cascade among white, Latinx, and Black participants, examining whether racial/ethnic differences vary by age, gender, or frequency of opioid use.

## Methods

### Study population

We examined the naloxone care cascade using baseline study visit data from an ongoing cohort of people who use illicit opioids in NYC (*N* = 575) aged 18 years or older, recruited between April 2019 and April 2020 using respondent-driven sampling [31, 32]. Baseline data were collected via a face-to-face interview. Levels of naloxone training and access during non-using and using days were assessed. The baseline assessment compensated eligible participants $60 for the approximate 2.5-h visit, which included urinalysis and overdose prevention and naloxone training and provision. Upon completion of baseline assessment, participants were trained in Overdose Education and Naloxone Distribution (OEND). The training included a 12-min NYC Department of Health training video and provision of a take-home naloxone kit. All study activities were approved by the New York University Grossman School of Medicine Institutional Review Board.

### Measures

#### Race/ethnicity

Participants were asked about both race and ethnicity. We first asked: “Do you consider yourself to be Hispanic or Latino/a?” Participants were then asked to endorse the race with which they identified. Following the US Census categorizations used to monitor diversity when combining race and ethnicity, we categorized participants as Hispanic or Latinx (“Latinx” used heretofore to be concise, *n* = 229), non-Hispanic white (*n* = 105), non-Hispanic Black (*n* = 217), or another race (*n* = 24). We first examined the naloxone care cascade in the sample overall, and then measured racial/ethnic differences in white, Latinx, and Black participants due to adequate sample to support reliable sub-group estimates.

#### Naloxone cascade outcomes

*Naloxone training* Participants were asked “Have you ever been trained in overdose prevention and response and received naloxone (Narcan), the medication to reverse an opioid-related overdose, before entering the study?”. Those who responded affirmatively were coded as having received training in naloxone administration (yes versus no).

#### Current naloxone possession and past 30-day daily naloxone access

Among all participants, regardless of naloxone training, we asked, “Do you currently have a naloxone kit?” Those who responded they currently have a naloxone kit were categorized as *currently possessing naloxone* (yes versus no). We also asked, “On how many of the last 30 days did you have naloxone/Narcan available to you for use? Those who indicated they had naloxone on 30 of the last 30 days were categorized as having past 30-day *daily naloxone access* (yes vs no) during using and non-using days.

#### Past 30-day 100% access to naloxone and complete protection

The frequency of opioid risk behavior was measured using the Opioid Risk Behavior Scale (ORBS) [17]. One goal of the ORBS scale is to calculate the number of illicit opioid use events within the past 30-days in which there was naloxone present in the room and another person to administer the medication. To do so, the average daily number of illicit opioid use events reported by participants is multiplied by the reported average number of days, out of a 30 day period, on which opioids were used. The resulting estimate was then shared with participants; if they deemed it incorrect, participants were able to input their own estimates (*n* = 7 participants elected to correct the estimate, which was used in analyses). Participants were then asked to estimate (a) the number of such events at which naloxone was available; and (b) the number of events at which both naloxone and someone else were present. Those who had naloxone in the room during 100% of opioid using events in the past 30-days were categorized as having *100% naloxone access during opioid use* (yes versus no). Those who had naloxone and someone else present at 100% of opioid use events within the past 30-days were categorized as having *complete protection* (yes vs no).

#### Factors associated with naloxone access

To contextualize potential differences in naloxone care cascade levels by race/ethnicity, we examined racial/ethnic differences in a range of socioeconomic, social support, opioid use indicators demonstrated to affect naloxone access, based on the extant literature. Socioeconomic indicators included gender (male, female); age, dichotomized at the median (≤ 50 vs > 50 years); current employment; current homelessness; and prior history of incarceration. Support factors included cohabitation with a romantic partner, whether received emotional support in the past 3 months from someone in the respondent’s network of people who use drugs, and whether received emotional support in the past 3 months from someone in the respondent’s network of people who do not use drugs. Opioid use indicators included opioid use disorder (OUD) severity measured using the Diagnostic and Statistical Manual of Mental Disorders Version 5 (DSM 5) criteria [33] and categorized as mild (score 2–3), moderate (score 4–5), or severe (score ≥ 6); OUD frequency in the past 30-days, with number of opioid use events dichotomized at the median (≤ 75 vs > 75 events); heroin injection in the past 30-days; regular (≥ 3 times/week at least monthly) drug injection for nonmedical purposes; syringe service program (SSP) utilization in the past 3 months; and drug stigma measured using six items adapted from the Stigma Consciousness Scale, on which participants reported, using a Likert-type response scale, their level of agreement with statements such as, “I worry that other people might find out that I use drugs”; we summed responses to the six items (range 0–24, alpha = 0.76) and dichotomized at the sample median (scores ≥ 13).

### Data analysis

All data analysis was conducted in Stata 16*.* We examined the univariable distribution of socioeconomic, social support, and opioid use characteristics in the overall sample and tested for differences by race/ethnicity (Pearson’s chi-squared test). We measured the prevalence of naloxone care cascade indicators in the total sample (*n* = 575), and, using logistic regression, measured unadjusted odds ratios (ORs) and adjusted odds ratios (AOR) and 95% confidence intervals (CIs) for associations between Black race and Latinx ethnicity (versus white, the referent), adjusting for gender, age (≤ 50 vs > 50 years), and past 30-day opioid use frequency (≤ 75 events versus > 75 events). We additionally assessed race differences in naloxone cascade indicators, stratified by gender (adjusted for age and opioid use frequency), age (adjusted for gender and opioid use frequency), and frequency of opioid use (adjusted for age and gender).

## Results

Socioeconomic, social support, and opioid use characteristics by race/ethnicity.

In the overall sample, 65.9% of participants were male, with slightly higher levels of being male among Latinx (70.7%) and Black (66.4%) participants than their white counterparts (53.3%) (Table 1). Overall, approximately half (50.8%) were 51 years or older, with significant racial/ethnic differences in the prevalence of being in this older cohort (Black: 74.7%, Latinx: 40.6%, white: 22.9%) (Table 1). A substantial proportion of the overall sample reported currently being unemployed (77.2%), homeless (30.3%), and four in five had a history of incarceration (80.2%). Rates of unemployment and incarceration were comparable by race/ethnicity, while homelessness was lower among Black (24.9%) and Latinx (30.1%) than white participants (41.9%). Overall, approximately one-quarter reported living with a committed partner (26.1%) and the majority received emotional support from the participants’ using network (64.5%) and non-using network (75.7%), with levels comparable across racial/ethnic groups. Overall, just over three-fourths had severe OUD with higher levels observed among white (82.9%) versus Latinx (74.2%) and Black (76.5%) participants. Elevated frequency of opioid using events was slightly higher among Latinx (54.6%) versus white (46.7%) and Black (43.8%) participants. Past 30-day heroin injection and past three-month syringe exchange program (SEP) utilization were much more commonly reported by white participants (injection 67.6%; SEP use 44.8%) than Latinx (injection 37.6%; SEP 35.8%) and Black (injection 14.4%; SEP use 15.7%). Approximately half scored a 13 or higher on the drug stigma scale overall, with Latinx participants slightly more likely to have elevated drug stigma (53.7%) than Black (44.7%) and white (48.6%) participants.

**Table 1:** Participant socioeconomic, support, and opioid-using characteristics among people who use opioids in New York City overall and stratified by race (*N* = 575)

	Overall (%)	White *n* (%)	Black *n* (%)	Latinx *n* (%)	Chi-square test result (*P* value) for participant characteristics by race
Gender					16.59 (0.035)
Female	190 (33.0)	47 (44.8)	72 (33.2)	65 (28.4)	
Male	379 (65.9)	56 (53.3)	144 (66.4)	162 (70.7)	
Missing	6 (1.0)	2 (1.9)	1 (0.5)	2 (0.9)	
Age	48.3 (11.27)				92.33 (0.000)
≤ 50 Years	283 (49.2)	81 (77.1)	55 (25.4)	136 (59.4)	
> 50 Years	292 (50.8)	24 (22.9)	162 (74.7)	93 (40.6)	
Employment					4.39 (0.821)
Unemployed	444 (77.2)	74 (70.5)	173 (79.7)	178 (77.7)	
Employed	124 (21.6)	30 (28.6)	41 (18.9)	48 (21.0)	
Missing	7 (1.2)	1 (1.0)	3 (1.4)	3 (1.3)	
Currently homelessness					12.28 (0.015)
No	401 (69.7)	61 (58.1)	163 (75.1)	160 (69.9)	
Yes	174 (30.3)	44 (41.9)	54 (24.9)	69 (30.1)	
Ever incarcerated					1.39 (0.709)
No	114 (19.8)	25 (23.8)	40 (18.4)	44 (19.2)	
Yes	461 (80.2)	80 (76.2)	177 (81.6)	185 (80.8)	
Lives with romantic partner					2.87 (0.579)
No	425 (73.9)	83 (79.1)	154 (71.0)	171 (74.7)	
Yes	150 (26.1)	22 (21.0)	63 (29.0)	58 (25.3)	
Using network gives emotional support					7.81 (0.452)
No	202 (35.1)	37 (35.2)	80 (36.9)	79 (34.5)	
Yes	371 (64.5)	68 (64.8)	137 (63.1)	148 (64.6)	
Missing	2 (0.4)	0 (0)	0 (0)	2 (0.9)	
Non-using network gives emotional support					10.62 (0.224)
No	133 (23.1)	23 (21.9)	48 (22.1)	54 (23.6)	
Yes	435 (75.7)	82 (78.1)	168 (77.4)	169 (73.8)	
Missing	7 (1.2)	0 (0)	1 (0.5)	6 (2.6)	
OUD severity					11.84 (0.159)
Mild or moderate	95 (16.5)	8 (7.6)	40 (18.4)	43 (18.8)	
Severe	440 (76.5)	87 (82.9)	166 (76.5)	170 (74.2)	
Missing	40 (7.0)	10 (9.5)	11 (5.1)	16 (7.0)	
Opioid use frequency (past 30-days)					36.85 (0.000)
≤ 75 events	293 (51.0)	56 (53.3)	122 (56.2)	104 (45.4)	
> 75 events	281 (48.9)	49 (46.7)	95 (43.8)	125 (54.6)	
Missing	1 (0.2)	0 (0)	0 (0)	0 (0)	
Ever regularly (≥ 3 times/week at least monthly) injected drugs for nonmedical purposes					110.07 (0.000)
No	33 (5.7)	3 (2.9)	12 (5.5)	18 (7.9)	
Yes	263 (45.7)	88 (83.8)	53 (24.4)	108 (47.2)	
Missing	279 (48.5)	14 (13.3)	152 (70.1)	103 (45.0)	
Any heroin injection (past 30-days)					106.54 (0.000)
No	365 (63.5)	32 (30.5)	185 (50.7)	136 (59.4)	
Yes	195 (33.9)	71 (67.6)	28 (14.4)	86 (37.6)	
Missing	15 (2.6)	2 (1.9)	4 (1.8)	7 (3.1)	
Visited SEP program (past 3 months)					44.30 (0.000)
No	347 (60.4)	4 (41.9)	164 (75.6)	123 (53.7)	
Yes	169 (29.4)	47 (44.8)	34 (15.7)	82 (35.8)	
Missing	59 (10.3)	14 (13.3)	19 (8.8)	24 (10.5)	
Elevated Stigma Score					13.47 (0.097)
0–12	277 (48.2)	52 (49.5)	114 (52.5)	96 (41.9)	
13+	280 (48.7)	51 (48.6)	97 (44.7)	123 (53.7)	
Missing	18 (3.1)	2 (1.9)	6 (2.8)	10 (4.4)	

### Naloxone care cascade

**Fig. 1:**
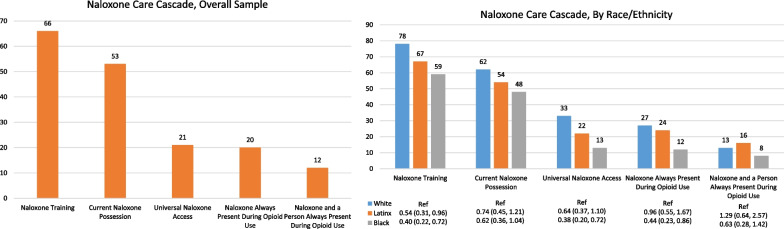
The naloxone care cascade in the overall sample (left) and stratified by race/ethnicity (right) bars indicate percentage endorsing each naloxone indicator. Odds ratios (ORs) and 95% confidence intervals (Cis) for race/ethnicity-naloxone associations are adjusted for younger age (< 50 years), gender, and lower opioid use frequency (≤ median (75) number past 30 day opioid use events)

Nearly two-thirds of people who use opioids were trained to administer naloxone (65.7%) and approximately half (52.5%) currently possessed naloxone, while a minority (20.9%) had past 30-day daily access during using and non-using days (Fig. 1). All participants who possessed naloxone had been trained in naloxone, and out of those who were not trained no person reported currently possessing naloxone. In the past 30-days during opioid use, 20.4% had 100% naloxone access and 12.4% had 100% complete protection (naloxone access and someone present to administer it).

### Naloxone training, by race/ethnicity

**Fig. 2:**
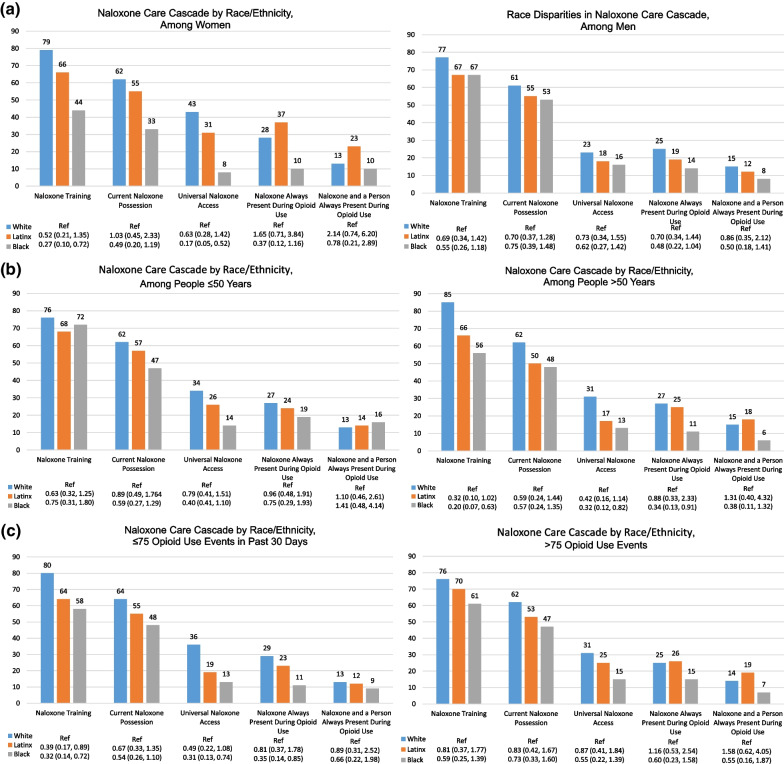
**a** The naloxone care cascade stratified by race/ethnicity among women (left) and men (right). Bars indicate percentage endorsing each naloxone indicator. Odds ratios (ORs) and 95% confidence intervals (CIs) for race/ethnicity-naloxone associations are adjusted for younger age and lower opioid use frequency. **b** The naloxone care cascade stratified by race/ethnicity among people ≤ 50 years old (left) and those > 50 years (right). Bars indicate percentage endorsing each naloxone indicator. Odds ratios (ORs) and 95% confidence intervals (CIs) for race/ethnicity-naloxone associations are adjusted for gender and lower opioid use frequency*.* **c** The naloxone care cascade stratified by race/ethnicity among people with lower (left) and higher (right) frequency opioid use in past 30 days. Bars indicate percentage endorsing each naloxone indicator. Odds ratios (ORs) and 95% confidence intervals (CIs) for race/ethnicity-naloxone associations are adjusted for younger age and gender

Levels of naloxone training were higher in white (78.1%) versus Latinx participants (67.3%; AOR for Latinx vs white comparison 0.54, 95% CI 0.31, 0.96) and Black participants (59.0%; AOR 0.40, 95% CI 0.22–0.72) (Figs. 1, 2a–c). The Black versus white racial disparity in naloxone training was particularly stark among women (Black 44%, White 79%; AOR 0.27, 95% CI 0.10–0.72), among people who are 51 years or older (Black 56%, White 85%; AOR 0.20, 95% CI 0.07–0.63), and among people with less frequent use based on lower than the median number of opioid using events per month (Black 58%, White 80%; AOR 0.32, 95% CI 0.14, 0.72).

### Current naloxone possession, by race/ethnicity

Currently possessing naloxone was higher in white participants (61.9%) than in Latinx participants (53.7%; 0.74, 95% CI 0.45–1.21) and Black participants (47.5%; AOR 0.62, 95% 0.36–1.04) (Figs. 1, 2a–c). Racial disparities did not appear to differ significantly by gender, age, or opioid use frequency.

### Past 30-day daily access to naloxone (non-using and using days), by race/ethnicity

Having daily access to naloxone was higher in white participants (33.3%) than in Latinx (21.8%; AOR 0.64, 95% 0.37–1.10) and Black (13.4%; AOR 0.38, 95% 0.20–0.72) participants (Figs. 1, 2a–c). The Black versus white racial disparity in having daily access appeared to be stronger among women (Black 8%, White 43%; AOR 0.17, 95% CI 0.05–0.52), among people who use opioids who are 51 years or older (Black 13%, White 31%; AOR 0.32, 95% CI 0.12–0.82), and among people with lower than the median number of opioid using events per month (Black 13%, White 36%; AOR 0.31, 95% CI 0.13–0.74).

### Past 30-day 100% naloxone access during opioid use, by race/ethnicity

Having naloxone present at 100% of opioid using events in the past 30-days also was comparable and higher in white (26.7%) and Latinx participants (24.0%; AOR 0.96, 95% CI 0.55–1.67) compared with Black participants (12.4%; AOR 0.44, 95% CI 0.23–0.86) (Figs. 1, 2a–c). The Black: white racial disparity in having 100% naloxone access during use appeared to be stronger among participants aged 51 years or older (Black 11%, White 27%; AOR 0.34, 95% CI 0.13–0.91), who had lower than the median number of opioid using events per month (Black 11%, White 29%; AOR 0.35, 95% CI 0.14–0.85), and in women, though this difference was not significant at the 0.05 level (Black 10%, White 28%; AOR 0.37, 95% CI 0.12–1.16).

### Past 30-day complete protection during opioid use, by race/ethnicity

Having 100% complete protection during opioid use also was comparable in White (13.3%) and Latinx participants (15.7%; AOR 1.29, 95% CI 0.64–2.57), while Black participants appeared to have lower levels of protection (8.3%; AOR 0.63, 95% CI 0.28, 1.42) (Figs. 1, 2a–c). The racial disparity did not appear to be concentrated in groups defined by gender, age, or opioid use frequency. It is notable that while 100% naloxone access was observed in 28% of white women and 25% of white men, there was a notable drop off in levels of 100% complete protection which was observed in 13% of white women and 14% of white men.

## Discussion

This study indicates there are significant racial/ethnic disparities in naloxone training, possession, and use during opioid use among people who use illicit opioids in NYC. While we observed gaps in naloxone access and use in the sample overall, dramatic racial/ethnic disparities in access to naloxone across the cascade were observed. Black participants were much less likely than white counterparts to be trained in naloxone administration, with Black women at particular risk given just over 40% had received training. Latinx participants also were less likely to be trained, possess, and have naloxone during use than white participants. This study highlights the ongoing need for improved access to harm reduction among drug users and the need for mixed methods research to best identify the barriers to access to and uptake of naloxone in distinct racial/ethnic groups. To advance overdose prevention across groups, there is a critical need for improved inclusion of Black, Latinx, and other persons of color in the leadership of both research and harm reduction policy and program development, to ensure overdose prevention efforts best foster inclusion, provide social support, and address the underlying social factors that affect engagement in harm reduction [34].

The racial and ethnic disparities observed in the present study may result from multiple underlying causes. One NYC study found that neighborhoods with a significantly higher proportion of white residents compared to Black residents had more pharmacies with naloxone in stock [5]. Disparities in naloxone access also may be a result of historically lower access to harm reduction services and subsequently, naloxone in Black communities [9], given that naloxone training is often offered through harm reduction organizations. Indeed, we observed that, compared with their white counterparts, Black participants in our sample were both less likely to report drug injection and also less likely to report recent SEP access. Further, prior studies have highlighted the ‘double stigma’ of substance use and racism, negatively impacting the utilization of substance use treatment services [19]. While some early opposition to syringe services pointed to institutional racism and a failure to remediate drug-related harms in communities of color directly [35], more recent concerns have tended to highlight racial disparities in stigmatization, stereotyping, and criminal justice responses to drug use in Black communities, all of which create additional burden for, and inhibit acceptability of, harm reduction in Black communities [10, 36]. These urban naloxone “deserts” in disadvantaged neighborhoods exacerbate existing health disparities and disparities in drug-related mortality [37].

Our observation of significant gaps in naloxone access across racial/ethnic groups is consistent with findings from recent studies in Baltimore, California, and Massachusetts [22, 28, 38]. Disturbingly, only one in ten participants in our study had complete protection during opioid use, with significant gaps in protection observed among white as well as racial/ethnic minority participants. While over 60% of white participants reported currently possessing naloxone, just over one-quarter indicated naloxone was universally present during use, and 13% of white participants were fully protected by naloxone and a person present to administer it, highlighting the ongoing concern about solitary drug use as a driver of overdose risk even in the presence of naloxone. There is a need for research to better understand why these low levels of naloxone access persist even among the most advantaged groups and in a city that has invested a lot into harm reduction efforts. There is a critical need to better understand barriers to naloxone access for Black women, in particular. Given evidence that intentional drug overdose deaths have increased among Black women in the most recent reporting periods, we need to better understand the degree to which access, stigma, or intention explain lack of naloxone coverage among Black women, to best advance overdose prevention for this group [39].

The frequency of solitary use among study participants is a strong indication that interventions need to be developed and expanded to address solitary drug use [37]. The use of “buddy systems” and other peer-driven forms of support may hold promise and help better harness protective benefits of social networks of people who use drugs, especially if a network member is connected to a harm reduction agency or other location to obtain naloxone, which can greatly extend the reach of naloxone distribution while providing other forms of support [40, 41]. If having peer or other support on hand for each use event is not possible, at least in NYC people who use drugs can now take advantage of the recently sanctioned and above ground Overdose Prevention Centers (OPCs) that are operating in several of NYC neighborhoods. By providing a safe indoor environment for the use of illegal substances, by any mode of administration, both sanctioned and unsanctioned OPCs have been demonstrated to effectively remove the risk of OD fatalities by having staff and/or peers observe drug use with oxygen and naloxone handy immediately in the event of an overdose [42]. Whether supported by a peer or an OPC, it is more important that ever to be protected when using illicit drugs given increased overdose risk as a result of increased exposure to fentanyl in the drug supply, which is 50 times more potent than heroin [34, 43–45].

The study was limited by the somewhat small sample sizes due to comparisons across race/ethnicity categories and further stratification by additional factors. Importantly, modest sample size limited statistical power to detect significant associations, despite large point estimates suggesting the existence of differences. Further, small sample size prevented estimation of rates in other racial/ethnic groups which are significant minority populations in New York City, and assessment of specific subgroups of Latinx people despite evidence of significant heterogeneity within Latinx groups [46–48]. We are also limited by concerns with generalizability of findings to other settings, given New York City’s expansive naloxone distribution infrastructure, which is relatively unique compared with other US settings. Further, a second study using another recruitment method or in another US setting may observe different naloxone care cascades and different relationships with race/ethnicity. Finally, the study may be limited by measurement error, given our use of self-reported measures of opioid use and naloxone use; both recall and social desirability bias may have affected the validity of these measures. Nonetheless, the study provides evidence to suggest that important racial/ethnic differences in naloxone access exist and indicates these should be assessed in additional cohorts.

In conclusion, there remain considerable gaps in opioid overdose naloxone protection that need to be addressed by improving access to training, reducing barriers to keeping naloxone, and reducing the likelihood people who use opioids are using alone, across racial/ethnic groups. There is a desperate need for further research *within* racial/ethnic groups to investigate the resource, systemic racism, intersectional stigma, and social fragmentation factors driving gaps across the care cascade. In addition, tailored programs and structural changes should be investigated; these may include alternatives to police response to overdose events, such as novel, public health-oriented programs involving social worker response. Ultimately, we need to encourage people who use drugs, as well as their friends and family members, to carry naloxone. We also should encourage people who use drugs to use together and adopt norms related to taking turns and monitoring each other after drug administration, particularly when using a new or untested supply [37]. In the context of an ongoing public health crisis of opioid related morbidity and mortality, it is more important than ever that we redouble our efforts to equip people with naloxone and social support to address disparities in access.
